# Hemophagocytic Lymphohistiocytosis Secondary to Chronic Lymphocytic Leukemia Progression

**DOI:** 10.7759/cureus.34128

**Published:** 2023-01-24

**Authors:** Nina Jancar, Filipa Sousa Gonçalves, José Fragoso Duro, Patrício Aguiar, Catarina Jacinto Correia

**Affiliations:** 1 Internal Medicine, Hospital de Santa Maria, Centro Hospitalar Universitário Lisboa Norte (CHULN), Lisbon, PRT; 2 Medicine, Lisbon University, Lisbon, PRT; 3 Transfusion Medicine & Immunohemotherapy, Hospital de Santa Maria, Centro Hospitalar Universitário Lisboa Norte (CHULN), Lisbon, PRT

**Keywords:** fever, septic shock, hepatomegaly, hemophagocytic lymphohistiocytosis (hlh), b-cell chronic lymphocytic leukemia (b-cll)

## Abstract

Hemophagocytic lymphohistiocytosis (HLH) is an acute, rare systemic hyperinflammatory disorder caused by a dysregulated immune cell function and massive cytokine release, often leading to multiple organ involvement and failure. Fever, hepatosplenomegaly, cytopenia, elevated liver enzymes, hypertriglyceridemia, and hyperferritinemia are the hallmarks of the disease. Its primary (genetic) form is typically observed in pediatric patients and its secondary, acquired form is seen in adult patients with an underlying autoimmune, malignant, or infectious disease. It is not frequently reported in patients with chronic lymphocytic leukemia (CLL) without an infectious or pharmacological trigger. We present a case of a 71-year-old patient with hemophagocytic lymphohistiocytosis due to the progression of chronic lymphocytic leukemia.

## Introduction

Hemophagocytic lymphohistiocytosis (HLH) is a rare, potentially fatal acute systemic hyperinflammatory disorder caused by immune system overactivation [1-3]. Its primary form occurs in patients with underlying genetic defects and is diagnosed primarily in the pediatric population. Acquired HLH arises in patients with underlying autoimmune, malignant, or infectious diseases [3-7]. It has an estimated yearly incidence of 1/800000 people [3] but is probably underdiagnosed in adults due to the overlap of its symptoms with sepsis and malignancies [4-5]. The most common clinical manifestations include fever and splenomegaly [1]. However, multiple organ infiltration by activated T cells and macrophages leading to multiple organ failure is not uncommon [1-3,8]. Typical laboratory findings include pancytopenia, altered liver enzymes, hypertriglyceridemia, hyperferritinemia, and coagulopathy. The HLH-94 diagnostic criteria and HScore for reactive hemophagocytic syndrome help establish the diagnosis of HLH [2,8]. The mortality remains high, and the treatment is based on supportive and immunosuppressive therapy, pro-apoptotic chemotherapy, and the treatment of the underlying cause [2-4,7].

## Case presentation

We present a case of a 71-year-old woman with a medical history of chronic lymphocytic leukemia (CLL) stage Rai 0 Binet A diagnosed in 1997 (24 years before the hospitalization), previous* Pneumocystis jirovecii* infection, essential arterial hypertension, and hypothyroidism. She was medicated with levothyroxine 0.1mg, amlodipine 5mg, and olmesartan+hydrochlorothiazide 20mg + 12.5mg. She was not receiving any treatment for CLL.

**Figure 1:**
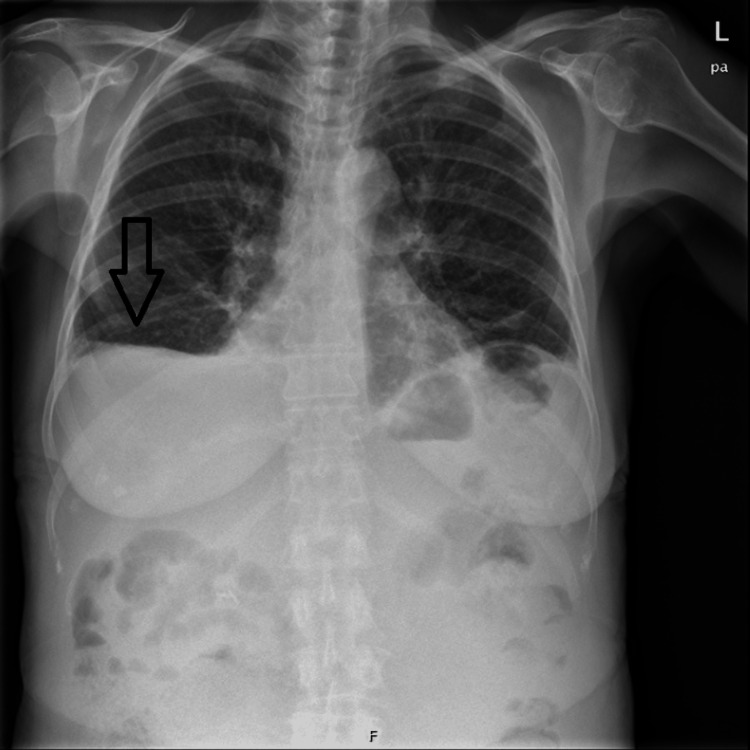
Chest radiograph at admission showing pleural effusion

The patient presented with fever, night sweats, exertional dyspnea, and nonproductive cough, which started six months before the hospitalization. Laboratory investigations at admission (Table 1) showed new onset pancytopenia (hemoglobin 7.3g/dL, leukopenia 3550/uL with 42% of lymphocytes and platelets 109000/uL), and elevation of C-reactive protein (CRP) (8.29mg/dL), liver enzymes (aspartate transaminase (AST) 242U/L, alanine transaminase (ALT) 125U/L, gamma-glutamyl transferase (GGT) 282U/L), and total bilirubin (1.3mg/dL). Peripheral blood immunophenotype showed monoclonal lymphocytosis compatible with B cell chronic lymphocytic leukemia (CLL-B). Pleural effusion was documented on the chest radiograph (Figure 1). 

**Table 1:** Laboratory tests at admission showing pancytopenia, the elevation of liver tests, and CRP CRP: C-reactive protein, AST: Aspartate transaminase, ALT: Alanine transaminase, GGT: Gamma-glutamyl transferase

Laboratory test	Value at admission	Normal range
Hemoglobin	7.3g/dL	12-15.3g/dL
Leukocytes	3550x10^9^	4000-11000x10^9^
Lymphocytes	1480x10^9^	1000-4800 x10^9^
Thrombocytes	109000x10^9^	150-450 x10^9^
CRP	8.29mg/dL	<0.5 mg/dL
AST	242U/L	0-32U/L
ALT	125U/L	0-33U/L
GGT	282U/L	0-40U/L
Total bilirubin	1.30mg/dL	<1.20mg/dL

**Figure 2:**
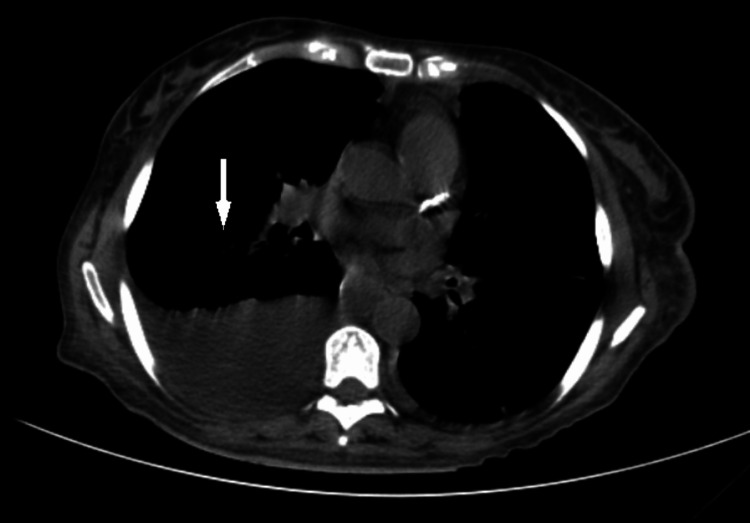
Lung CT showing bilateral pleural effusion

**Figure 3:**
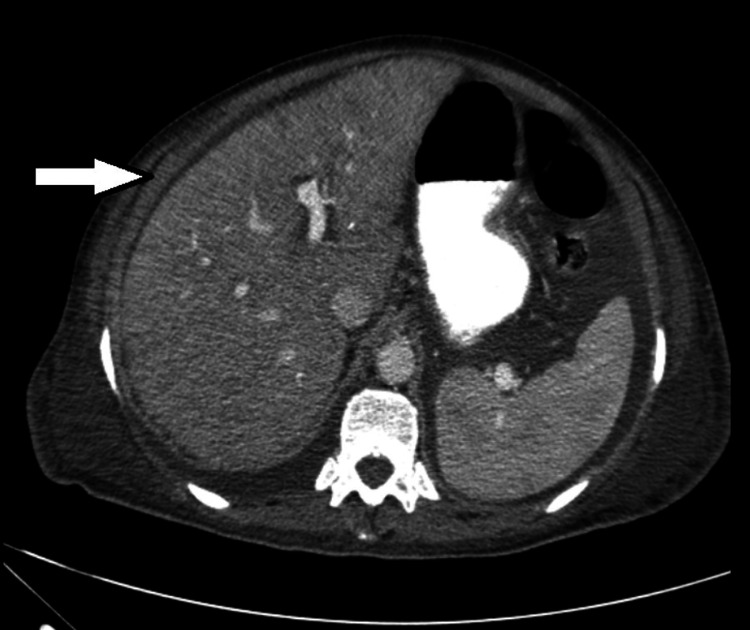
Abdominal CT scan showing hepatomegaly and ascites

**Figure 4:**
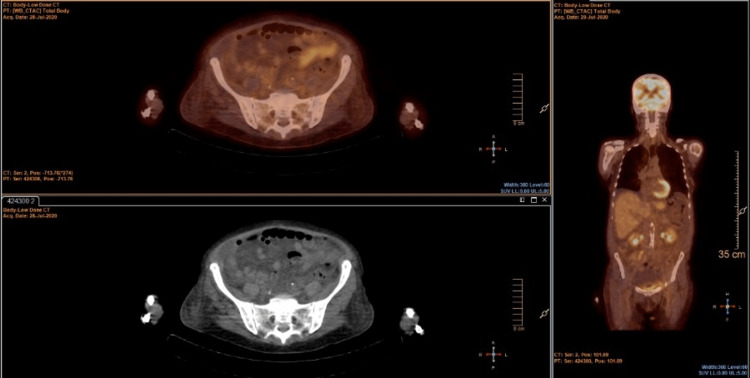
Positron emission tomography (PET) scan showing discrete metabolic activity in the bone marrow

**Figure 5:**
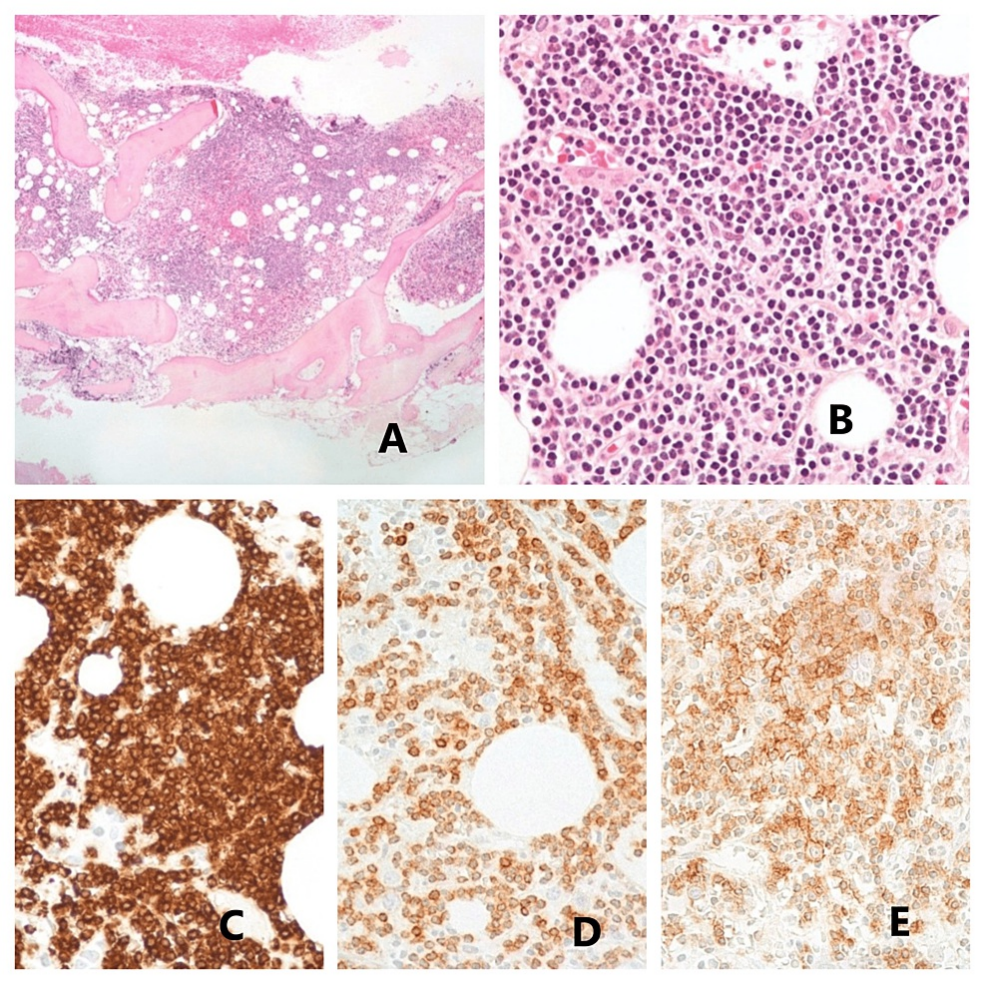
Microscopic examination of the bone marrow Seen is a moderately increased cellularity due to lymphoid cell proliferation with a sheet pattern (A). The lymphoid cells had round nuclei with irregular nuclear membranes, dense chromatin, and scarce cytoplasm (B). In the histochemical study, these cells showed diffuse immunoreactivity for the cluster of differentiation (CD)79a (C) with co-expression of CD5 (D) and CD23 (E), compatible with infiltration by chronic lymphocytic leukemia (CLL). Photos courtesy of Dr. Tiago Oliveira (Hospital de Santa Maria, CHULN, Lisbon, Portugal)

Further diagnostic tests were performed. Bone marrow biopsy documented moderate leukemic infiltration. A whole-body CT scan showed bilateral pleural effusion (Figure 2), mediastinal and hilar adenopathy, hepatomegaly, and ascites (Figure 3). A positron emission tomography (PET) scan revealed discrete metabolic activity in the bone marrow (Figure 4), compatible with the bone marrow biopsy findings (Figure 5). 

**Figure 6:**
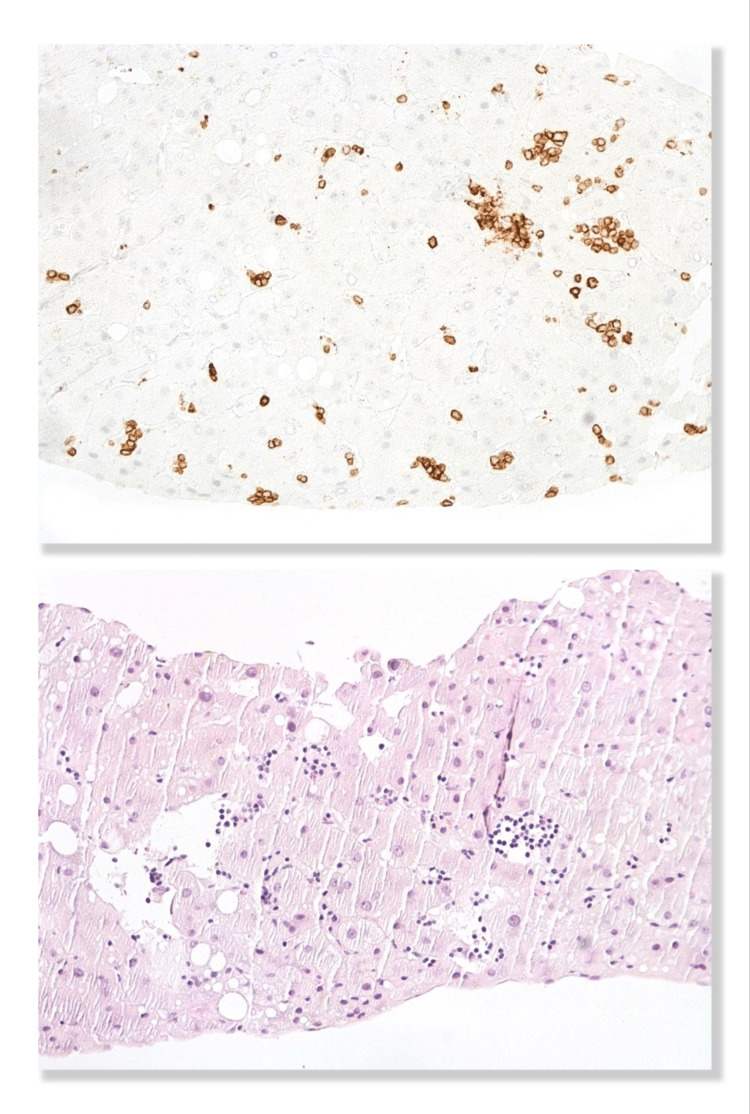
Microscopic analysis of the liver biopsy shows mild macro and microvacuolar steatosis and moderate perisinusoidal fibrosis (A), as well as sinusoidal infiltration by small lymphoid cells (B), identical to the ones observed in the bone marrow with the same diffuse immunoreactivity for the cluster of differentiation (CD)79a. Photos courtesy of Dr. Tiago Oliveira (Hospital de Santa Maria, CHULN, Lisbon, Portugal)

Pleural fluid analysis was compatible with an exudate: the effusion protein/serum protein ratio was 0.6 and the effusion lactate dehydrogenase (LDH)/serum LDH ratio was 0.71. However, a cytologic exam and immunophenotype of the pleural fluid were not done due to insufficient sample quantity. Paracentesis was also performed, documenting a serum ascites albumin gradient (SAAG) >1.1g/dL, suggestive of portal hypertension, and 250 leukocytes with 54.7% of mononucleated cells on the cytologic exam; no neoplastic cells were observed. In addition, a transjugular liver biopsy was performed, and leukemic infiltration of the liver was documented, confirming the diagnosis of CLL progression (Figure 6). 

Due to persistent fever, worsening pancytopenia, and progressive liver enzyme elevation, HLH was suspected. Further laboratory investigations (Table 2) revealed hyperferritinemia (13097/uL), hypofibrinogenemia (92mg/dL), natural killer (NK) cell depletion, and elevation of soluble cluster of differentiation (CD)25 (>5000/uL). Therefore, an HScore for reactive hemophagocytic syndrome of 251 points was calculated [7]. 

**Table 2:** Laboratory tests showing aggravated pancytopenia and liver enzymes, altered coagulation tests, negative infectious serologies/cultures, IGRA, and autoimmune profile INR: International normalized ratio, aPTT: Activated partial thromboplastin time, IGRA: Interferon-gamma release assay, AST: Aspartate transaminase, ALT: Alanine transaminase, CD25: Cluster of differentiation 25, CMV: Cytomegalovirus, EBV: Epstein-Barr virus

Laboratory test	Value	Normal range
Hemoglobin	6.5g/dL	12-15.3 (g/dL)
Leukopenia	2468x10^9^	4000-11000x10^9^
Thrombocytopenia	62000x10^9^	150000-450000/uL
AST	197U/L	0-32U/L
ALT	119U/L	0-33U/L
Total bilirubin	1.75mg/dL	<1.20mg/dL
Ferritin	13000/uL	13-150ng/mL
Fibrinogen	92mg/dL	200-400mg/dL
Soluble CD25	>5000pg/mL	325-1785pg/mL
INR	1.34	-
aPTT	40 sec	29 sec
IGRA	Negative	-
CMV	Negative	-
EBV	Negative	-
HIV	Negative	
Blood and urine culture	Negative	-
Autoimmune profile	Negative	-

Infectious causes (including bacterial infection, tuberculosis, human immunodeficiency virus (HIV), cytomegalovirus (CMV), and Eppstein-Barr virus (EBV) infection), autoimmune and pharmacological causes, were excluded as the potential triggers, and thus CLL progression was assumed as the etiology of the HLH.

The patient was started on dexamethasone, etoposide, and rituximab after a hematology consultation, resulting in the resolution of the fever, improvement of cytopenias, as well as normalization of coagulation, and liver enzymes. Nevertheless, the patient developed febrile neutropenia and respiratory sepsis with multiple organ dysfunction. Despite the antibiotic therapy with piperacillin/tazobactam and amikacin, aggravation with septic shock ensued. The patient was transferred to an intensive care unit, where she passed away shortly after due to refractory septic shock.

## Discussion

Hemophagocytic lymphohistiocytosis is an acute systemic hyperinflammatory disorder caused by a defect in granule-mediated cytotoxicity, characterized by persistent T cell and macrophage activation, leading to massive cytokine release known as "cytokine storm" [1-3,6-7]. It is a rare disease characterized by a rapidly progressive clinical course and a high mortality rate with a median survival of about two months [3,9]. Its primary form is typically diagnosed in pediatric patients and is associated with mutations of genes involved in lymphocyte cytotoxicity [1]. The secondary, reactive form is observed principally in adult patients with an underlying autoimmune (systemic lupus erythematosus (SLE), systemic sclerosis, rheumatoid arthritis, adult-onset Still disease), malignant (lymphoma, T cell, and natural killer (NK) cell leukemia), or infectious disease (CMV, EBV, HIV, herpes simplex virus (HSV), tuberculosis, malaria). It can also be observed in patients with severe burns and those undergoing major surgery or organ transplant [3,7,10].

Common clinical manifestations include fever, hepatosplenomegaly, serous cavity effusion, and central nervous system (CNS) symptoms. With the progression of the disease, multiple organ infiltration by the activated T cells and macrophages occurs, ultimately leading to organ damage and the corresponding clinical manifestations [9]. Laboratory hallmarks of HLH are bi or pancytopenia, elevated liver enzymes, hypertriglyceridemia, elevated ferritin, hypofibrinogenemia, elevated soluble CD25, and low or absent NK cell activity [3,10,11].

Hemophagocytic lymphohistiocytosis should be suspected in a patient with a fever and multiple organ involvement, which can't be attributed to any other apparent cause [3]. It can pose a diagnostic challenge, especially in patients with malignancies, as the symptoms can be easily attributed to the underlying disease [5], as seen in our case. 

The association of HLH with chronic lymphocytic leukemia is not frequently reported in the literature [12-13], especially without an accompanying infectious, autoimmune or pharmacological trigger, all of which were excluded in our patient. Therefore, CLL progression documented during the hospitalization was assumed to be the etiology of HLH. 

The diagnosis of HLH was based on the HLH-2004 criteria i.e., the patient presented six out of eight criteria: persistent fever, peripheral pancytopenia, hyperferritinemia, hyperfibrinogenemia, diminished NK cell activity, and an elevated soluble CD25. An HScore for reactive hemophagocytic syndrome of 251 points was calculated, meaning that the probability of hemophagocytic syndrome was superior to 99% [14].

A prompt diagnosis and treatment are essential since the disease is associated with a poor prognosis [8,11]. Treatment of the underlying trigger, suppression of the dysregulated immune response, and supportive treatment are the cornerstones of treatment [3,8,11,15]. The HLH-94 protocol consists of eight weeks of induction therapy with dexamethasone and etoposide, followed by continuation therapy with dexamethasone, etoposide, and cyclosporine, which is indicated in the case of the relapse of the disease or primary HLH, as a bridge to hematopoietic stem cell transplant [3,8,11,15]. The protocol was revised in 2004 and advocated the addition of cyclosporine in the induction phase [3,15]. However, the studies did not show significant improvement in the outcomes of the disease, and because of cyclosporin-associated toxicities and contraindications, the HLH-94 protocol remains the recommended standard of care [15]. Long-term immunosuppressive treatment is not advisable due to an elevated risk for serious infectious complications [1], especially in elderly and frail patients [16]. 

After a hematology consultation, our patient was started on dexamethasone, etoposide, and rituximab. Unfortunately, it was impossible to promptly begin treatment of CLL (which was the underlying trigger in this case) with ibrutinib due to pending authorization from the hospital's pharmacy and therapeutics committee. Despite the initial clinical and laboratory improvement, the patient developed severe infectious complications associated with albeit a short course of immunosuppressive therapy that proved to be as fatal as untreated HLH. 

## Conclusions

Hemophagocytic lymphohistiocytosis is an acute hyperinflammatory disease associated with a poor prognosis. The association of HLH with the progression of chronic lymphocytic leukemia is not frequently described, especially in the absence of an underlying infectious or pharmacological trigger. The diagnosis is challenging since the clinical manifestations are not specific and can be attributed to the underlying hematological malignancy. It should be suspected and ruled out in patients with prolonged fever and multiple organ dysfunction, given the high mortality rate in untreated patients. The authors would like to emphasize the challenges of managing the complications associated with immunosuppressive therapy, especially in elderly and frail patients, which can be as fatal as HLH. 
